# Sensory Neuronopathy Revealing Severe Vitamin B12 Deficiency in a Patient with Anorexia Nervosa: An Often-Forgotten Reversible Cause

**DOI:** 10.3390/nu9030281

**Published:** 2017-03-15

**Authors:** Jérôme Franques, Laurent Chiche, Stéphane Mathis

**Affiliations:** 1 Hôpital de La Casamance, 13400 Aubagne, France; j.franques@hp-lacasamance.fr; 2 Service de Médecine Interne, Hôpital Européen, 13003 Marseille, France; 3 Service de Neurologie, CHU Bordeaux 33000 Bordeaux, France; stephane.mathis@chu-bordeaux.fr

**Keywords:** sensory neuropathy, cobalamin deficiency, vitamin B12, anorexia nervosa

## Abstract

Vitamin B12 (B12) deficiency is known to be associated with various neurological manifestations. Although central manifestations such as dementia or subacute combined degeneration are the most classic, neurological manifestations also include sensory neuropathies. However, B12 deficiency is still rarely integrated as a potential cause of sensory neuronopathy. Moreover, as many medical conditions can falsely normalize serum B12 levels even in the context of a real B12 deficiency, some cases may easily remain underdiagnosed. We report the illustrating case of an anorexic patient with sensory neuronopathy and consistently normal serum B12 levels. After all classical causes of sensory neuronopathy were ruled out, her clinical and electrophysiological conditions first worsened after folate administration, but finally improved dramatically after B12 administration. B12 deficiency should be systematically part of the etiologic workup of sensory neuronopathy, especially in a high risk context such as anorexia nervosa.

## 1. Introduction

Vitamin B12 (B12) or cobalamin deficiency is a real public health issue, since it has been reported to occur in up to 20% of the elderly and up to 30% in specific subgroups of patients above 70 years [1,2]. The most characteristic manifestations of severe deficiency are haematological (i.e., macrocytic anemia) and central neurological manifestations such as reversible dementia or subacute combined degeneration [3]. However, peripheral neuropathies account for 30% to 50% of the neurological symptoms associated with B12 deficiency [4]. They are mostly symmetrical, length-dependent axonal sensory and sometimes sensorimotor neuropathies [5]. To date, B12 deficiency has not been included among the classical causes of sensory neuronopathies, which usually have an acute onset, are sometimes asymmetrical, or have a non-length-dependent distribution [6]. Importantly, the fact that B12 serum levels assessment can be falsely normalized by various medical conditions (including acute and chronic liver diseases, often of alcoholic origin, various neoplasias, malignant hemopathies such as myeloproliferative diseases, and renal insufficiency), a situation often termed as “functional B12 deficiency”, may have participated to the underdiagnosis of this association [7,8]. Indeed, in the cases where the serum levels are in the normal range but the index of suspicion for a B12 deficiency remains strong, the diagnosis should rely on either surrogate biomarkers for B12 deficiency (high homocysteine or methylmalonic acid (MMA) levels, medullary aspiration findings) and/or on the improvement under B12 supplementation. However, in practice, these additional investigations are rarely performed [8].

We report here an illustrating case of sensory neuronopathy in the context of chronic anorexia with dramatic improvement after vitamin B12 treatment.

## 2. Case Presentation

A 30 year-old female patient, with no past medical history, presented with painful paresthesia in the feet, which extended within two weeks to the hands and lips. She had lost 10 kg during the last four months in a context of anorexia nervosa. Clinical examination revealed only skin hyperpigmentation. The neurological examination showed the existence of diffuse areflexia and hypoesthesia in all four limbs. No proprioceptive ataxia or weakness was detected. The biological tests showed low plasma folate level (vitamin B9) (4.5 nmol/L for normal value > 7 nmol/L) with normal serum B12 or vitamin E levels. The electrophysiological study was in favor of a severe non-symmetrical, non-length-dependent sensory neuropathy involving all four limbs, with an electro-clinical pattern of sensory neuronopathy (Table 1). The motor nerve conduction study was normal, without slowing of nerve conduction velocities or any conduction block, and the evoked motor potentials (studied in all four limbs using transcranial magnetic stimulation) were also normal. 

The cerebrospinal fluid analysis (cytology, protein and glucose levels, immunoelectrophoresis) and brain/spinal cord MRI findings were normal. The whole-body CT-scan was normal. The immunological tests including anti-neuronal, anti-ganglioside, anti-SSA/SSB were negative. The salivary gland biopsy was normal. No monoclonal gammopathy or immunoglobulin light chain was found. The HIV blood test was negative, as other usual serologies (EBV, CMV, hepatitis, Lyme disease); *Treponema pallidum* particle agglutination assay (TP-PA) and venereal disease research laboratory test (VDRL) turned out to be negative. 

The patient was given vitamins B1, B6, and B9 at usual doses. Cutaneous symptoms responded well to this treatment, but she was admitted to hospital three months later because of a subacute proprioceptive ataxia causing frequent falls. Owing to her persistent state of anorexia, she had then lost 10 more kg. She was still suffering from painful paresthesia in wrists and ankles despite continuous treatment with vitamins B1, B6, and B9. She was only able to walk a few meters unaided. At physical examination, no pallesthesia was observed at levels of the knees. No motor symptoms or pyramidal sign were detected. She presented no orthostatic hypotension nor other signs of dysautonomia. The folate serum level was then normalized (34 nmol/L) and B12 level still in the normal range (314 pmol/L, normal value > 220 pmol/L), but hyperhomocysteinemia (25 µmol/L; normal value < 15 µmol/L) was observed (without renal insufficiency), as well as macrocytosis (mean corpuscular volume (MCV): 102 fl, normal value < 100 fl) without anemia (complete blood count was strictly normal), suggestive of B12 deficiency. Anti-transglutaminase, anti-endomysium, and intrinsic factor/gastric parietal cell antibodies were negative and oesogastroduodenal fibroscopy was normal. 

The patient was therefore given daily 1000 µg intramuscular injections of B12 for two months. Although anorexia persisted, this treatment significantly decreased painful paresthesia within three months in parallel of a normalization of homocysteinemia and MCV. Five months later, her ability to walk was no longer restricted. At last examination, nine months after B12 administration, there was no more symptom in the upper limbs, and pallesthesia was quite normal in the feet with normal walking. Electrophysiological tests confirmed a progressive increase of the amplitude of sensory nerve action potential (SNAP) with time (Table 1). 

## 3. Discussion and Conclusions

Vitamin B12 deficiency is usually associated with various hematological, gastrointestinal and neurological/neuropsychiatric disorders [3]. Hematological manifestations may include anemia with a macrocytic blood picture and megaloblastic bone marrow, as well as isolated macrocytosis, as in our patient, and even in the complete absence of hematological manifestations in some patients with clear neurological manifestations [5,9]. Indeed, neurological manifestations may be the earliest and often the only manifestation of B12 deficiency [9]. 

While the main neurological complication is peripheral neuropathy, subacute combined degeneration, and optic nerve neuropathy can occur. Other rare manifestations include dementia, cerebellar ataxia, leukoencephalopathy, orthostatic tremor, myoclonus, ophthalmoplegia, catatonia, vocal cord paralysis, syringomyelia-like distribution of motor and sensory deficits, and autonomic dysfunction. Clinical and pathological involvement of the peripheral nervous system has been well described with B12 deficiency, usually with electrophysiological abnormalities suggestive of a length-dependent sensorimotor axonopathy [5].

We describe here a severe case of ataxia due to a sensory neuronopathy that was dramatically improved after B12 administration in a patient suffering severe anorexia nervosa. Because of the known poor sensitivity of the radio-immunological methods to detect it, a functional B12 deficiency was suspected because of the clinical context and in spite of repeated normal blood serum B12 levels [7]. Indeed, from measured total serum B12, only a small and variable proportion of B12 is active, so that metabolic B12 deficiency is common in patients with a serum B12 in the reference range [10]. To be reasonably sure that total serum B12 is adequate, some have proposed to consider as correct only levels above ~400 pmol/L [11,12]. Especially below that cut-off, as in our case, confirmatory tests are needed to assess adequacy of functional B12: measurement of methylmalonic acid, or in folate-replete patients, total homocysteine [8]. The worsening under folate therapy was also evocative of the process termed the ‘folate trap’ [13]. Indeed, folates and B12 both act as cofactors in the process of DNA synthesis. Giving folates to patients with B12 deficiency may mobilize the remaining stocks of B12 required to synthesize the nucleic acids on which fast cell replacement processes depend. This mobilization of the last remaining B12 supplies may occur to the detriment of other biochemical reactions involving B12, such as those resulting in the formation of methionine and hence that of myelin. Ultimately, a therapeutic test with B12 supplementation may be mandatory to confirm that B12 deficiency is the cause of the observed neurological manifestations. Importantly, this test may be worthwhile even in patients with an alternative cause of peripheral neuropathies. In a recent study, Solomon observed that 83% of 78 patients with neuropathy (including 35 patients with other known causes of neuropathy) improved after having been treated with oral or parenteral B12 [14]. Although patients with severe B12 deficiency with severe clinical complications first need parenteral B12, oral B12 is usually adequate to achieve target levels [15], so that as long as anorexia persists, patients should be given oral B12 supplements in the long term.

Sensory neuronopathies are characterized by the primary involvement of sensory neurons in the dorsal root ganglia [16]. New diagnostic criteria for sensory neuronopathies have been published [5,17]. Using these criteria, our patient was classified in the ‘possible sensory neuronopathy’ group. Indeed, the electrophysiological study including 14 sensory nerves showed an asymmetrical and a more pronounced involvement of the proximal sensory nerves (cutaneous median and lateral forearm from both upper limb) compared to distal nerves (medial, radial and ulnar nerves) in favor of a non-length-dependent neuropathy. Finally, paresthesia in the lips is not a classical sensory localisation of length-dependent axonopathy. The known etiological causes of sensory neuronopathies are paraneoplastic, toxic, dysimmune, nutritional (lack of vitamin E, nicotinic acid, and riboflavin), infectious, and genetic causes. Approximately 50% of the cases of sensory neuronopathy are said to be ‘idiopathic’ [16], but B12 deficiency has not been included so far among the definite causes. Pathological findings on totally gastrectomized rats (a murine model for B12 deficiency) also support this causality. Indeed, it was reported that non-vasogenic edema present in endoneurium of the dorsal root ganglia disappeared in response to B12 administration [18]. 

In the case of our patient, there seems to have been a link between B12 deficiency and sensory neuronopathy: (a) the classical etiological patterns were ruled out; (b) the neurological symptoms worsened after folate administration (‘folate trap’); and (c) the clinical and electrophysiological findings improved dramatically in response to B12 treatment concomitantly with the normalisation of homocysteinemia and MCV. 

Vitamin B12 deficiency has not yet been recognized as one of the causes of sensory neuronopathy, mainly because of the poor sensitivity of radio-immunological methods of determination and/or by the absence of systematic screening in the presence of alternative concomitant causes. In patients at high risk such as those with anorexia nervosa, it is therefore essential to identify as quickly as possible this deficiency, even if B12 serum levels are normal or paradoxically high [19], since a specific treatment is available, the time elapsing before its introduction being the main prognostic factor.

## Figures and Tables

**Table 1:** Evolution of amplitude of sensory nerve action potential (SNAP) on three successive electrophysiological studies of sensory nerves: initial study (M0) before treatment, second study five months after (M5), then third study nine months after the vitamin B12 treatment initiation (M9).

		M0	M5	M9
right side	median nerve	4.4 µV	8.6 µV	11 µV
	ulnar nerve	6 µV	9.3 µV	10 µV
	radial nerve	5 µV	10 µV	11 µV
	cutaneous median forearm nerve	0 µV	7.5 µV	8 µV
	cutaneous lateral forearm nerve	0 µV	0 µV	4.3 µV
	sural nerve	0 µV	0 µV	3 µV
	fibular nerve	0 µV	0 µV	0 µV
left side	median nerve	4.6 µV	5.6 µV	9 µV
	ulnar nerve	NO	2.5 µV	6 µV
	radial nerve	5.3 µV	5.9 µV	11.4 µV
	cutaneous median forearm nerve	0 µV	0 µV	8 µV
	cutaneous lateral forearm nerve	0 µV	0 µV	1.4 µV
	sural nerve	0 µV	1.2 µV	6.4 µV
	fibular nerve	0 µV	0 µV	0 µV

## Abbreviations

B12	vitamin B12
CMV	cytomegalovirus
CT-scan	computerized tomography scanner
DNA	deoxyribonucleic acid
EBV	Epstein-Barr virus
HIV	human immunodeficiency virus
MRI	magnetic resonance imaging
SNAP	sensory nerve action potential
TP-PA	*Treponema pallidum* particle agglutination assay
VDRL	venereal disease research laboratory test
